# CD163+ perivascular macrophages in schizophrenia: a research framework for testing macrophage-related mechanisms

**DOI:** 10.3389/fpsyt.2026.1833449

**Published:** 2026-07-17

**Authors:** Hans C. Klein, Paul C. Guest, Michael E. Benros, Chavit Tunvirachaisakul, Christian Scheiber, Michael Maes, Robert Yolken, Karl Bechter, Johann Steiner

**Affiliations:** 1 Department of Nuclear Medicine and Molecular Imaging, University of Groningen, Groningen, Netherlands; 2 Department of Research and Education, Addiction Care Northern Netherlands, Groningen, Netherlands; 3 Department of Psychiatry, Otto-von-Guericke-University Magdeburg, Magdeburg, Germany; 4 Laboratory of Translational Psychiatry, Otto-von-Guericke-University Magdeburg, Magdeburg, Germany; 5 Laboratory of Neuroproteomics, Department of Biochemistry and Tissue Biology, Institute of Biology, University of Campinas (UNICAMP), Campinas, Brazil; 6 Copenhagen Research Centre for Biological and Precision Psychiatry, Mental Health Centre Copenhagen, Copenhagen University Hospital, Copenhagen, Denmark; 7 Department of Psychiatry, Faculty of Medicine, Chulalongkorn University, Bangkok, Thailand; 8 Center of Excellence in Cognitive Impairment and Dementia, Faculty of Medicine, Chulalongkorn University, Bangkok, Thailand; 9 Department of Neurology, Ulm University Hospital, Ulm, Germany; 10 Mental Health Center, University of Electronic Science and Technology of China, Chengdu, China; 11 Research Institute, Medical University of Plovdiv, Plovdiv, Bulgaria; 12 Department of Psychiatry, Medical University of Plovdiv, Plovdiv, Bulgaria; 13 Cognitive Impairment and Dementia Research Unit, Faculty of Medicine, Chulalongkorn University, Bangkok, Thailand; 14 Division of Stanley Developmental Neurovirology, Department of Pediatrics, Johns Hopkins School of Medicine, Baltimore, MD, United States; 15 Clinic for Psychiatry and Psychotherapy II, Ulm University, Guenzburg, Germany; 16 Center for Behavioral Brain Sciences (CBBS), Magdeburg, Germany; 17 Center for Health and Medical Prevention (CHaMP), Magdeburg, Germany; 18 German Center for Mental Health (DZPG), Partner Site Halle-Jena-Magdeburg, Magdeburg, Germany; 19 Center for Intervention and Research on Adaptive and Maladaptive Brain Circuits Underlying Mental Health (C-I-R-C), Halle-Jena-Magdeburg, Magdeburg, Germany

**Keywords:** Bacille Calmette–Guérin, blood-brain barrier, CD163, herpes simplex virus type 1, hypothesis testing, perivascular macrophages, schizophrenia, trained immunity

## Abstract

Elevated densities of CD163+ perivascular macrophages have been reported in schizophrenia post-mortem brain tissue, particularly in regions involved in neurodevelopment, dopaminergic signaling, and blood–brain barrier (BBB) regulation. However, the biological significance and developmental lineage of these findings remain unclear. While CD163 is linked to regulatory and scavenging functions, macrophage activation states form a continuum and cannot be inferred from any single marker. This Perspective outlines a structured, testable research framework to determine whether this accumulation reflects altered responsiveness to persistent intracellular, inflammatory, systemic, or treatment-related signals. To test this, we propose sequential methodological aims, including defining CD163+ cell localization and phenotypes in predefined brain regions, and assessing viral and non-viral molecular signals using spatial transcriptomic and cell-specific methods. This framework also involves comparing macrophage activation states across schizophrenia and other psychiatric and non-psychiatric control groups, using single-cell and single-nucleus sequencing. By not presuming a specific infectious aetiology, this approach will provide a general methodology to investigate macrophage-related mechanisms across diverse potential triggers. Within this model, HSV-1 is evaluated strictly as an illustrative proof-of-concept candidate for testing intracellular pathogen responses, rather than an exclusive cause, as epidemiological associations have been inconsistent and localization of viral materials within these cells has not been demonstrated. Similarly, Bacille Calmette–Guérin (BCG)-associated trained immunity is introduced strictly as a preliminary, ex vivo/*in vitro* approach to probe macrophage reprogramming and plasticity. Ultimately, this framework provides a systematic approach for investigating macrophage-related mechanisms and their potential drivers in schizophrenia without presupposing a specific underlying aetiology.

## 1 Introduction

Schizophrenia is increasingly recognized as a disorder involving interactions between neurodevelopmental, vascular, and immune processes (1, 2). Epidemiological and experimental studies have linked prenatal and early-life infections, maternal immune activation, chronic inflammation, and altered blood–brain barrier (BBB) function to schizophrenia risk (2, 3). Although the precise mechanisms remain unclear, these observations have contributed to growing interest in immune-cell populations located at critical neurovascular interfaces, including a potential role in modulating neurogenesis in specialized brain niches (4, 5) and circuit-wide disruptions in dopaminergic signaling (6, 7). This neurogenic/niche-related interpretation should be treated cautiously because adult human neurogenesis remains more debated than in rodent models.

Recent post-mortem studies have identified an accumulation of CD163+ perivascular macrophages (PVMs) in brain regions implicated in schizophrenia (2, 8–12), consistent with alterations at the neurovascular interface. Several mechanisms could account for this, including BBB disruption (11), peripheral immune cell recruitment (12, 13), treatment-related effects (14, 15) and responses to infections (16, 17), persistent microbial products, or other stimuli (18, 19). Beyond acute triggers, prolonged exposure to microbial signals or latent infections could induce sustained alterations in macrophage behavior.

Latent neurotropic viral infections such as HSV-1 represent one candidate mechanism because this virus can interact with myeloid cells (20–25). Other viruses, including cytomegalovirus (CMV) and SARS-CoV-2, have also been reported to infect or interact with monocytes and macrophages (26–29). However, epidemiological studies examining associations between HSV-1 or CMV exposure and schizophrenia have produced inconsistent findings (30–32). Furthermore, direct and reproducible localization of HSV-1 or other viruses to defined brain cell types in schizophrenia, including neurons, glia, endothelial cells, microglia, or CD163+ PVMs, has not been demonstrated. Existing evidence is based largely on serological studies, cognitive associations, and indirect epidemiological observations rather than cell-type-specific detection in brain tissue. Alternatively, any associations between viral exposure, schizophrenia-related phenotypes, and increased CD163+ PVM density may result from broader immune-related processes rather than localized infection within the brain.

Trained immunity provides a potential framework for understanding how transient stimuli can induce persistent reprogramming of myeloid cells. Bacille Calmette–Guérin (BCG) is discussed solely as a well-characterized experimental model of this state (33, 34), not as evidence that BCG affects schizophrenia. Although trained immunity mechanisms have been studied primarily in peripheral monocytes and macrophages, any relevance to central nervous system (CNS)-associated macrophages remains poorly understood.

In this Perspective, we propose a structured and testable hypothesis that CD163+ macrophage accumulation may reflect a state of altered immune responsiveness to persistent intracellular stimuli in a subset of schizophrenia patients. Importantly, this hypothesis does not assume a specific causal agent. Instead, it outlines a framework for evaluating potential mechanisms, including involvement of viruses such as HSV-1. Similarly, BCG-induced trained immunity is used solely as an experimental platform to probe myeloid cell plasticity. Our aim is to provide a transparent roadmap that distinguishes established findings from untested assumptions and to outline the experimental steps required to test the proposed model.

## 2 Established findings

### 2.1 CD163+ macrophages in the CNS and schizophrenia

CD163+ PVMs are a conserved component of the CNS immune system and are present under both physiological and pathological conditions. These cells are typically located in Virchow-Robin/perivascular spaces and contribute to immune surveillance, antigen presentation, and vascular homeostasis (35–38). Under physiological conditions, they are present at relatively low densities (39), whereas post-mortem studies suggest increased densities in selected brain regions in subsets of individuals with schizophrenia (40). Depending on physiological and pathological state, CD163+ PVMs may derive from embryonically established progenitors and/or be replenished by peripheral monocytes under inflammatory conditions (35–38).

Post-mortem studies have reported increased densities of CD163+ macrophages in specific brain regions in subsets of individuals with schizophrenia. However, similar cell populations are also observed in other conditions such as multiple sclerosis (41), Alzheimer’s disease (42), tuberculosis (TB) (43), human immunodeficiency virus (HIV) (37), herpes simplex virus (HSV) infections (44), and possibly in mood disorders including major depression and bipolar disorder (45, 46) (**Supplementary Table**). These widespread observations suggest that CD163+ macrophages represent a shared immune response pathway rather than a disease-specific feature.

### 2.2 Functional interpretation of CD163 expression

CD163 expression is often used as a marker associated with anti-inflammatory or regulatory macrophage profiles, including interleukin (IL)-10 and transforming growth factor (TGF)-β–signalling (47–51). However, this association is context-dependent and does not define a discrete or stable functional state. Increasing evidence indicates that macrophage phenotypes exist along a dynamic spectrum, influenced by local microenvironments (52, 53).

In the CNS, this complexity is further amplified by differences between resident microglia and infiltrating monocyte-derived macrophages and region-specific factors. Therefore, the presence of CD163+ cells should be interpreted cautiously, and any functional conclusions will require experimental validation.

### 2.3 Macrophage responses to intracellular stimuli

In peripheral tissues, macrophages can adopt functional states that influence their ability to respond to intracellular pathogens and chronic inflammatory signals. In TB, chronic infection is associated with a shift toward CD163+ macrophages that permit pathogen persistence (54, 55). In HSV-1 infections, macrophage maturation can be impaired, and CD163-expressing or regulatory macrophage phenotypes may favor viral persistence (24, 44, 56). Conversely, more pro-inflammatory activation states may enhance pathogen control but carry the risk of tissue damage.

These principles provide a general framework for considering how macrophage functional states might influence disease processes. However, their applicability to brain-resident macrophages, PVMs, or other CNS-associated macrophage populations in schizophrenia requires further exploration.

### 2.4 Immune training and macrophage plasticity

Trained immunity is included in this Perspective as a potential experimental procedure for assessing macrophage plasticity, not as evidence that BCG can affect schizophrenia. BCG vaccination can induce trained immunity in peripheral innate immune cells and can promote epigenetic and transcriptional reprogramming in myeloid progenitors, which may enhance subsequent innate immune responses (57–59). Reported effects include altered production of cytokines such as interleukin (IL)-1β, IL-6, and tumor necrosis factor (TNF)-α, as well as changes in monocyte and macrophage responsiveness to secondary challenges.

While these effects are well documented in peripheral immune compartments, their relevance to CNS-resident or CNS-infiltrating macrophages is not established. Although peripheral monocytes can enter the CNS and differentiate into macrophage-like cells under inflammatory conditions (35–38), the extent to which BCG-induced changes affect CNS immune function or CNS-associated macrophage status remains unknown.

## 3 Critical gaps and testable questions

Despite the observations outlined above, several questions remain unanswered and represent essential next steps for interpreting mechanisms of action and before considering clinical translation.

### 3.1 Presence or absence of viral material in CD163+ macrophages

To date, no study has convincingly demonstrated the presence of viral material (e.g., HSV-1 DNA, RNA, or proteins) within accumulated CD163+ PVMs in schizophrenia. Addressing this question will require sensitive, cell-specific, and orthogonal approaches.

For example, peripheral blood mononuclear cells (PBMCs) could be isolated from the peripheral blood of patients and controls, and CD163+ monocytes enriched by fluorescence-activated cell sorting (FACS). Isolated cells could then be assessed for HSV-1 DNA by PCR (60) and HSV-1-encoded miRNAs by qPCR (11, 61). Since latent or low-copy viral detection is technically challenging, the findings should be validated using complementary approaches such as digital droplet PCR (62) and targeted sequencing (63).

In another approach, post-mortem brain tissue studies could examine CD163+ PVMs using immunohistochemical co-localization of CD163 and viral markers (64), RNA *in situ* hybridization (65), targeted RNA sequencing approaches (66), or spatial transcriptomics approaches (67). It would also be informative, if pathogen-related signals are identified, to determine whether such signals are present in peripheral monocyte or progenitor compartments, while distinguishing true infection from carriage, phagocytosed material, or contamination (68–70).

### 3.2 Specificity and function of CD163+ macrophage accumulation in the CNS

It remains unclear whether the accumulation of CD163+ macrophages in schizophrenia reflects a disease-specific process or a more general response to factors such as BBB dysfunction, systemic inflammation, medication, or other environmental factors.

Recent single-cell and single-nucleus transcriptomic studies have found subtle widespread immune and myeloid transcriptional alterations in schizophrenia, mostly characterized by changes in inflammatory or stress-response pathways rather than shifts in cell proportions or abundance (71–75). Furthermore, these findings suggest that these alterations are heterogeneous across individuals and may be restricted to particular cellular states or patient subgroups.

Comparative analyses across psychiatric and neurological disorders will also be necessary to determine specificity of CD163+ macrophage accumulation. In addition, it remains unknown whether these cells exhibit altered responsiveness to inflammatory or infectious stimuli, and whether such responses are consistent across patients or confined to specific subgroups. Single-cell and functional assays will be required to define these states more precisely, including transcriptomic profiling, cytokine response assays, ex vivo stimulation experiments, and analysis of cell-state changes across disease subgroups.

### 3.3 Modifiability of macrophage functional states

If altered macrophage states are identified, it still remains to be determined whether they represent stable, biologically meaningful phenotypes or transient responses to environmental signals. While trained immunity provides a conceptual framework for understanding how prior exposures can lead to persistent functional changes in myeloid cells (34, 76), applicability to CNS-associated PVMs has not been established, and most evidence has been derived from peripheral monocytes.

Given these uncertainties, the immediate objective is to determine whether distinct macrophage states exist, how these arise, and whether they are associated with viral exposure, inflammatory signaling, or other processes. Accordingly, we propose a stepwise experimental framework in which each stage provides a clear decision point for determining whether further investigation is warranted. Such an approach should emphasize rigorous hypothesis testing, including attempts to falsify the proposed mechanism, and prioritize reducing key unknowns. Importantly, any persistence of trained immune states may be beneficial, detrimental, or neutral depending on the surrounding microenvironment and functional consequences, underscoring the need for direct experimental assessment.

## 4 BCG-trained immunity as an experimental probe

At the present stage, BCG should be considered as an experimental probe of trained immunity in preclinical models to test whether macrophage responsiveness, antigen presentation, cytokine production, or pathogen-related molecular readouts can be altered. Evidence from TB biology, cancer immunology, and trained immunity studies indicates that BCG can induce memory-like responsiveness in myeloid cells (34, 77–80). However, these findings are largely derived from peripheral immune systems and it remains unknown if similar effects occur in CNS PVMs.

Examples such as HSV-associated erythema multiforme and hematogenous HSV models illustrate that viral material can interact with or be transported by myeloid lineages under certain conditions (68, 81–83). Similarly, HIV/SIV models have demonstrated that viruses can be disseminated via monocyte or macrophage transport into the CNS (84, 85). These examples are given only as precedents for immune cell trafficking and do not provide evidence for viral involvement in CD163+ macrophage accumulation in schizophrenia.

Accordingly, this Perspective is intended as a neutral experimental approach for testing whether CD163+ macrophage states are associated with infectious, inflammatory, or other immune-related triggers in schizophrenia (5, 8–10, 86, 87) and whether such macrophage states are associated with immune alterations in patient subgroups (88, 89). At this stage, this should be regarded only as a working model to help structure further research and does not assume the presence of intracellular pathogens.

**Figure 1:**
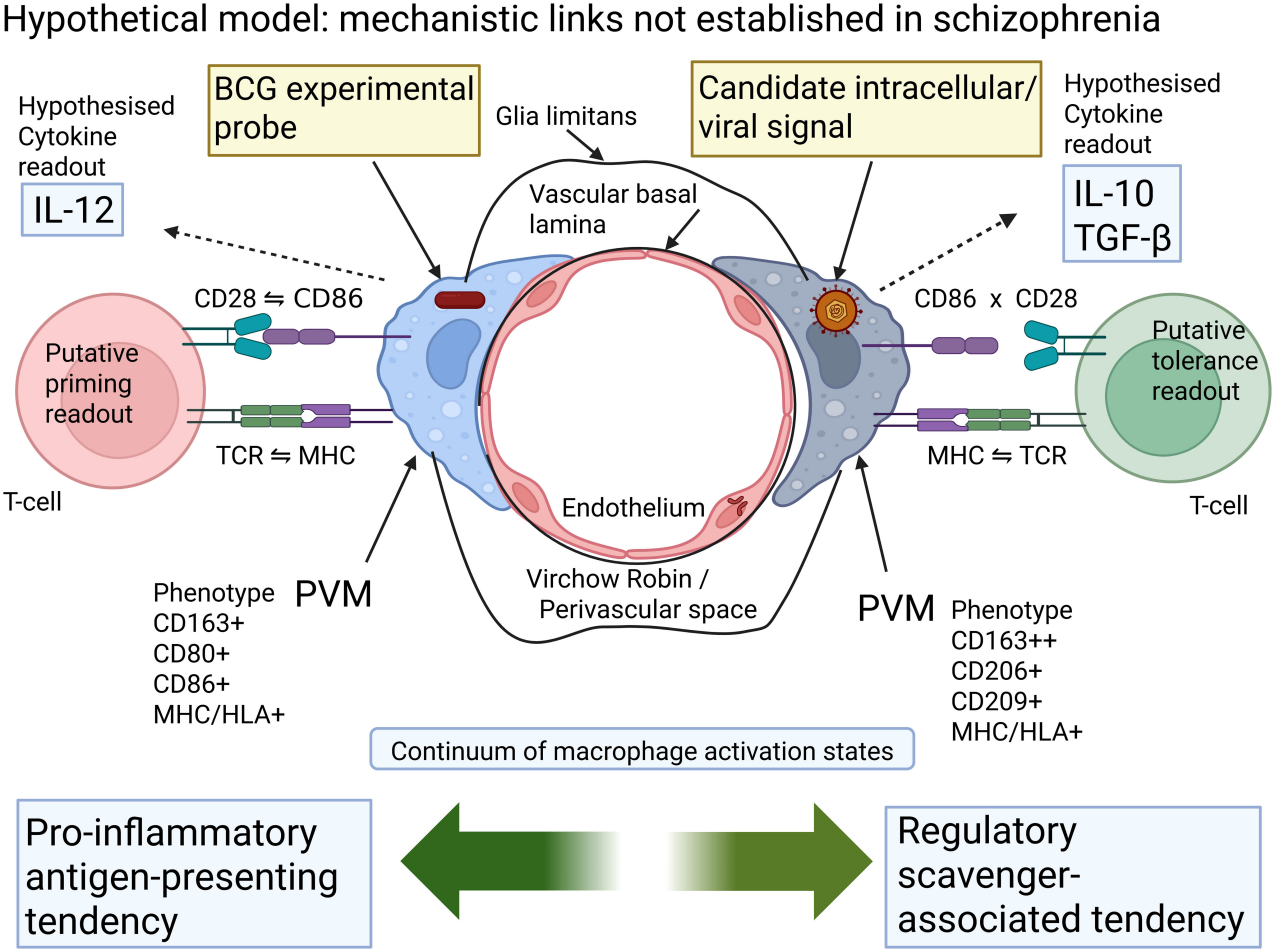
Conceptual and testable framework for investigating CD163+ PVM accumulation in schizophrenia. The figure presents a hypothetical research framework in which persistent intracellular, inflammatory, or other biological signals may contribute to macrophage responsiveness at the vascular-CNS interface. Solid black arrows indicate anatomical or cellular relationships represented in the conceptual model. Dashed arrows indicate hypothesized readout directions or unvalidated mechanistic links. Directionality does not imply established causality in schizophrenia. Candidate PVM phenotypes expressing CD163 are illustrated using co-stimulatory receptors (CD80/CD86) and scavenger-associated receptors (CD206/CD209). “+/++” notation, where shown, indicates relative marker expression within the conceptual model and not measured abundance or validated activation states. Cautionary note: All depicted pathogen-related processes and immune-cell interactions are hypothetical and require experimental validation. Candidate viral or intracellular signals are shown only as illustrative stimuli and not as causative factors in schizophrenia. Macrophage states are represented as a continuum rather than discrete phenotypes, and BCG-associated trained immunity is depicted solely as an experimental probe of macrophage plasticity, not as evidence for a potential intervention in schizophrenia. CD, cluster of differentiation; HSV-1, herpes simplex virus type 1; IL, interleukin; PVM, perivascular macrophage; TGF-beta, transforming growth factor beta. Created in BioRender. Klein, H. (2026) https://BioRender.com/2xpzjl2.

**Figure 2:**
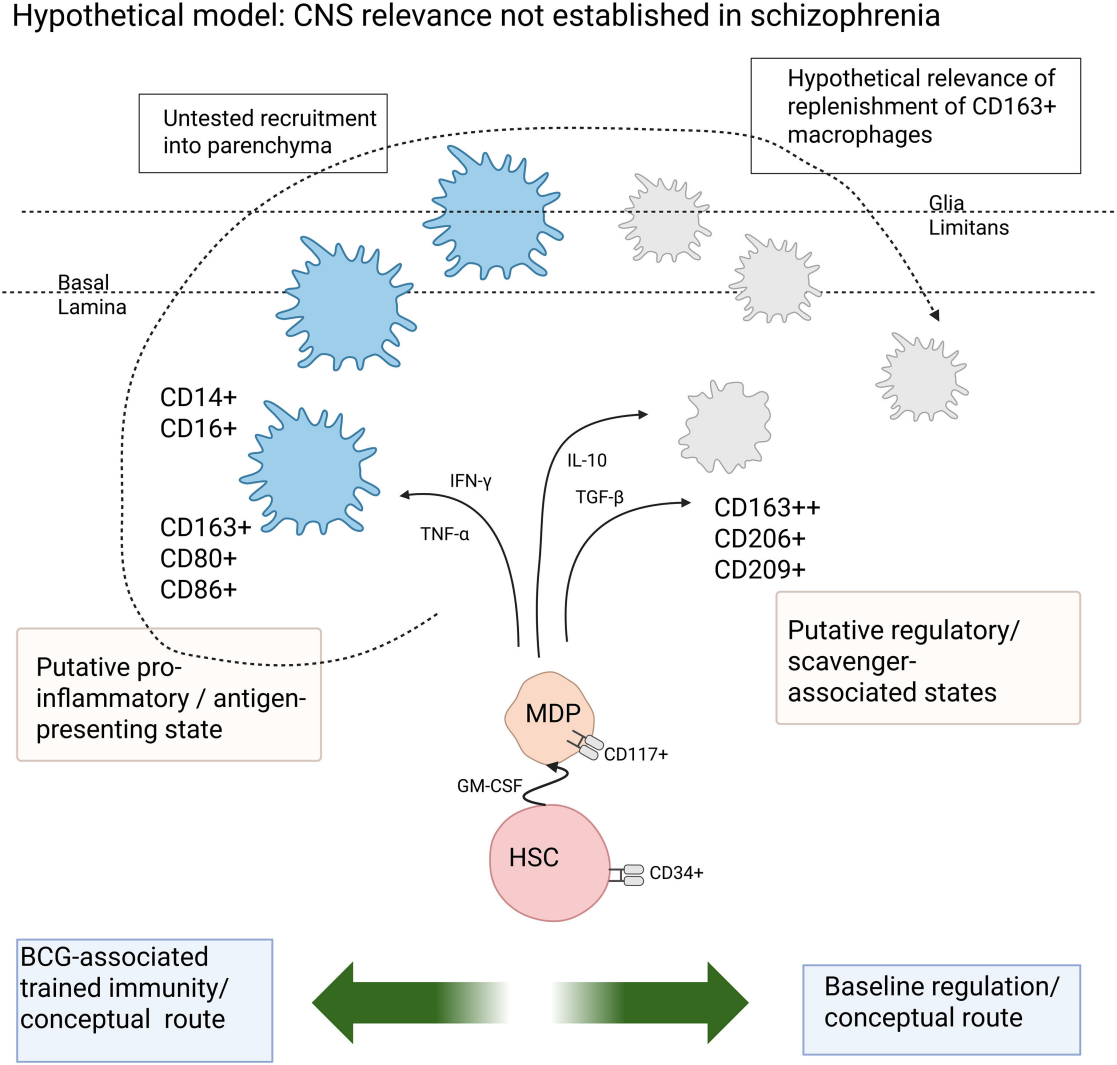
Established peripheral trained immunity pathways and hypothetical applications for assessing macrophage plasticity in schizophrenia. The figure distinguishes established BCG-associated trained immunity effects in peripheral innate immune cells and hematopoietic progenitor compartments from untested hypotheses involving CNS-associated macrophages and CD163+ PVMs. BCG vaccination can induce reprogramming of peripheral innate immune and progenitor cells in experimental and clinical studies. In this conceptual model, hematopoietic stem cells give rise to monocyte-dendritic cell progenitors, which may differentiate toward cell states with relatively stronger pro-inflammatory/antigen-presenting features or toward regulatory/scavenger-associated features, depending on cytokine milieu and tissue context. Cautionary note: Whether peripheral trained immunity programs influence CNS macrophage recruitment, persistence, differentiation, or functional states in schizophrenia remains unknown. Accordingly, all CNS-directed pathways, CD163+ macrophage states, and proposed effects on neurovascular immune responses are hypothetical. “+/++” indicates relative marker expression within the conceptual model and does not imply measured abundance or validated activation states. This framework illustrates how trained immunity paradigms may be used to investigate macrophage plasticity and does not imply a role for BCG in schizophrenia treatment. Future studies should assess: (i) whether distinct CD163+ macrophage states are present in schizophrenia; (ii) whether these cells contain viral, bacterial, inflammatory, or other intracellular signals; (iii) whether findings can be replicated and validated; and (iv) whether trained immunity paradigms modify molecular, transcriptional, epigenetic, metabolic, or functional readouts in controlled ex vivo or *in vitro* systems. BCG, Bacille Calmette-Guerin; HSC, hematopoietic stem cell; GM-CSF: Granulocyte Monocyte Colony Stimulating Factor; IFN, interferon; IL, interleukin; MDP, monocyte-dendritic cell precursor; TGF-beta, transforming growth factor beta; TNF-alpha, tumour necrosis factor alpha. Created in BioRender. Klein, H. (2026) https://BioRender.com/fed1hqd.

A schematic overview of this tentative and testable framework is shown in **Figure 1**. In this model, BCG is depicted only as an experimental reference for testing myeloid-cell responsiveness and not as a schizophrenia treatment model. **Figure 2** illustrates established BCG-induced trained immunity in the periphery alongside untested extrapolations to CNS-associated PVMs. The CNS-directed components and CD163+ macrophage states are therefore hypothetical and require experimental validation.

## 5 Alternative explanations and limitations

The accumulation of CD163+ macrophages in the brains of individuals with schizophrenia is unlikely to reflect a single mechanism. Although persistent intracellular stimuli, including viral infections, are one possible explanation (16, 90), several alternative drivers and important limitations should also be considered.

First, pathogens other than HSV could contribute to altered macrophage activation states, including TB (43), HIV (37), and other viral, bacterial, or inflammatory stimuli. However, interpretations must be balanced against negative or neutral findings from large serological and molecular studies. Increased exposure to HSV-1, HSV-2, Epstein-Barr virus, or Toxoplasma gondii has not been consistently demonstrated after adjustment for confounders and most post-mortem studies have not supported an increase in CNS viral nucleic acids in schizophrenia (91, 92). These findings underscore the need for direct cell-specific validation in selected subgroups.

Second, disruption of BBB integrity may facilitate recruitment of peripheral monocytes into the CNS, where they differentiate into PVMs, including CD163+ cells (93–95).

Third, systemic inflammation seen in some individuals with schizophrenia may promote monocyte recruitment to the brain, such that CD163+ is macrophage accumulation reflects broad immunological status and not necessarily localized to the CNS (12, 13).

Fourth, antipsychotic medications may contribute to immune modulation, including modulation of cytokine profiles and macrophage/microglial activation states (14, 15).

Fifth, non-infectious inflammatory factors, such as tissue stress, vascular injury, metabolic disturbances, smoking, obesity, insulin resistance, and post-mortem variables, could also influence macrophage recruitment and differentiation independently of an infection (18, 19).

Several additional limitations constrain the present hypothesis. There is currently no direct evidence that HSV-1 is present within CD163+ PVMs in schizophrenia or that altered CD163+ macrophage states contribute causally to schizophrenia pathophysiology. Furthermore, peripheral monocyte findings may not directly translate to CNS-associated PVMs. Detection of latent or low-abundance viral material within low-abundance cell populations is technically challenging and requires stringent controls, orthogonal validation, and independent replication. Finally, although BCG-induced trained immunity has been demonstrated in humans, its effects on CNS-associated macrophages and any relevance to schizophrenia remain untested.

Together, these considerations suggest that CD163+ macrophage accumulation should be interpreted as a potentially convergent endpoint of multiple biological processes rather than evidence for a single causal pathway. Accordingly, the framework proposed here is intended to generate testable mechanistic questions rather than to establish causality or justify translational studies.

## 6 Rationale for stepwise testing of the hypothesis

Despite the uncertainties outlined above, the proposed framework provides a structured approach for investigating the unexplained biological findings. CD163+ macrophage accumulation in schizophrenia remains incompletely understood and warrants systematic studies such as those proposed here, regardless of whether infectious mechanisms are ultimately supported.

Even if the current hypothesis proves incorrect, systematic investigation of this finding may yield important insights into immune-related processes in the disorder. Second, the hypothesis is structured to be falsifiable because each assumption can be tested using sensitive and specific experimental approaches. Third, the framework is not restricted to a single causal factor and allows alternative explanations, including inflammatory, vascular, metabolic, or other biological drivers to be evaluated in the same experimental workflow.

Finally, increasing evidence suggests that schizophrenia is a heterogeneous disorder, with subgroups characterized by distinct immune profiles (96, 97). Investigating macrophage-related mechanisms and associated molecular signatures may therefore help clarify whether immune-related mechanisms contribute to specific patient subgroups and whether biological pathways associated with trained immunity have relevance in selected schizophrenia subgroups (98, 99).

### 6.1 Research framework

To systematically evaluate this model, we outline a series of four sequential experimental aims:

Aim 1: Map Spatial Distribution and Co-Marker Phenotypes. Quantify regional CD163+ cell abundance across schizophrenia, other psychiatric disorders, non-psychiatric neurological conditions, and healthy controls. Prioritize key neurovascular niches (subependymal, midbrain, and cortical regions) using multiplex panels to differentiate PVMs from resident microglia.

Aim 2: Interrogate Latent Pathogen and Host Inflammatory Signals. Screen affected microenvironments for low-abundance viral, bacterial, fungal, parasitic, or host interferon-response signatures. Isolate cell-specific material via laser-capture microdissection or cell sorting, and validate target signatures using orthogonal spatial techniques.

Aim 3: Profile Transcriptomic States via Single-Cell and Spatial Omic Platforms. Identify disease-associated cell states using single-cell/single-nucleus RNA sequencing, and map these profiles directly onto the physical neurovascular interface using spatial transcriptomics to define activation states via unbiased pathway analysis.

Aim 4: Validate Macrophage Modifiability in Functional Ex Vivo and *In Vitro* Models. Establish human cellular models using peripheral blood monocytes or induced macrophages from biomarker-stratified participants. Evaluate functional plasticity using standardized trained immunity assays (e.g., BCG stimulation) to quantify epigenetic, metabolic, and transcriptional reprogramming alongside cytokine, phagocytic, and pathogen-clearance dynamics.

## 7 Conclusion and future prospects

This Perspective presents a structured and testable framework to investigate the potential role of CD163+ macrophages in schizophrenia. The four-step framework outlined in Section 6.1 provides an operational roadmap for this approach. Although accumulation of these cells has been reported in subsets of patients, the functional significance and underlying drivers remain unclear. We propose that altered macrophage responsiveness to persistent intracellular or inflammatory stimuli represents a possible explanation for this observation, while emphasizing that current evidence does not establish the presence of specific pathogens, such as HSV-1, within CD163+ macrophages in schizophrenia, and that alternative mechanisms are also plausible.

The primary aim of this work is to guide stepwise mechanistic investigation. The key priorities include determining whether intracellular signals are present within CD163+ cells, characterizing their functional states, and establishing whether these states represent reproducible biological phenomena. Each stage provides opportunities for refutation or validation of the hypothesis.

If supported, this framework could contribute to a more refined understanding of immune heterogeneity in schizophrenia and help identify biologically-defined subgroups for further study. If not supported, the proposed approach will still help define the relevance and limits of macrophage-related processes in schizophrenia. Therefore, the value of this framework lies in its falsifiability and ability to reduce uncertainty through systematic testing rather than in any immediate translational implications.
