# Supplementary material for: CD163+ perivascular macrophages in schizophrenia: a research framework for testing macrophage-related mechanisms
Source: Front Psychiatry. 2026 Jul 17;17:1833449. doi: 10.3389/fpsyt.2026.1833449 (PMC13424148; doi:10.3389/fpsyt.2026.1833449)
Supplement: Supplementary file 1 [file Table1.docx]

**Supplementary Table. Selected evidence on CD163+ perivascular macrophages and related myeloid-cell markers relevant to macrophage hypotheses in schizophrenia**

| **Study** | **Markers / methods** | **Location / compartment** | **Key finding relevant to this Perspective** | **Interpretation and limits** |
| --- | --- | --- | --- | --- |
| Human post-mortem schizophrenia; high- vs low-inflammation subgroups and controls (Cai et al., 2020) | CD163 immunohistochemistry; immune-cell and endothelial/BBB-related mRNAs | Frontal cortex / brain parenchyma and perivascular interface | CD163+ macrophages were identified in brain parenchyma in >40% of high-inflammation schizophrenia brains; immune-cell/endothelial mRNAs were elevated in high-inflammation schizophrenia. | Directly relevant to the central observation. Supports subgroup-specific CD163+ macrophage accumulation but does not establish HSV-1, another pathogen, or BCG responsiveness. |
| Human post-mortem schizophrenia, particularly high-inflammation cortical subgroup (Zhu et al., 2022) | CD163, CD64, CD14, CCL2 and related macrophage/immune markers; CD163+ cells along blood vessels | DLPFC / prefrontal cortex; vascular-associated cells | Cortical studies support increased macrophage-associated signals in high-inflammation schizophrenia; CCL2 was increased and correlated with CD163 mRNA.. | Important replication/extension in cortex. Use as evidence for macrophage-associated changes in a subgroup, not as proof of a single mechanism or disease specificity. |
| Human post-mortem schizophrenia and bipolar disorder; inflammatory subgroups (North et al., 2021; Weissleder et al., 2021) | CD163, CD64, CD14, FCGR3A and ICAM1 mRNAs; microglial markers P2RY12/P2RY13; CD163+ cell density | Subependymal zone (SEZ) | High-inflammation schizophrenia showed increased peripheral immune-cell marker mRNAs and reduced microglia-related markers; CD163+ macrophage density was increased vs. low-inflammation controls. Weissleder et al. linked increased macrophages in the SEZ with reduced neurogenesis markers. | Directly relevant to SEZ findings. Adult human neurogenesis remains debated and findings are subgroup/region-specific. Does not prove pathogen-driven accumulation. |
| Human post-mortem schizophrenia, bipolar disorder, major depressive disorder, and controls (Mendez-Victoriano et al., 2024) | CD163+ cell density; CD163 protein; tyrosine hydroxylase (TH) mRNA | Substantia nigra and cerebral peduncles | Parenchymal CD163+ cell density was increased in the substantia nigra across major psychiatric disorders; CD163 protein was increased in schizophrenia; TH mRNA was reduced in schizophrenia and bipolar disorder and negatively correlated with parenchymal CD163+ cell density. | Supports relevance of CD163+ macrophage-like cells in dopaminergic regions but also shows lack of schizophrenia specificity. Use to broaden the framework beyond a schizophrenia-only mechanism. |
| Human post-mortem schizophrenia and controls; symptomatic-state and age effects considered (De Picker et al., 2021) | Microglial and PVM markers including IBA1, HLA-DR, CD68, CD64, CD16, CD206, CD163; CD3 T-cells | Prefrontal cortex | Study reported altered brain immune environment and increased Fc-gamma receptor-related expression but did not find recruited macrophages or increased CD163/CD206 PVM density overall. | Essential balancing row. Shows that CD163/PVM accumulation is not uniform across cohorts and supports the need for subgrouping, replication, and standardized phenotyping. |
| Human depressed suicides vs. controls (Torres-Platas et al., 2014) | IBA1 morphology; CD45 mRNA; MCP-1/CCL2; CD68 not significantly elevated | Dorsal anterior cingulate white matter; perivascular-associated myeloid signal | Reported evidence consistent with microglial priming and macrophage recruitment in depressed suicides, with increased IBA1/MCP-1-related signals. | Comparator only. CD163 was not directly measured. Include only to show that perivascular/myeloid changes are not unique to schizophrenia and need psychiatric comparison groups. |
| Human control CNS and multiple sclerosis tissue (Fabriek et al., 2005) | CD163; CD206/mannose receptor; MHC-II/HLA-DR; CD40; CD86 in control subset; CD80 and CD209/DC-SIGN in MS/inflamed context | Perivascular spaces in normal CNS; PVM and lesion-associated myeloid cells in MS | CD163 identified PVMs in normal human CNS, and CD163+ PVMs expressed antigen-recognition/presentation/co-stimulatory molecules. In inflamed MS tissue, CD163 was also detected in lesion-associated myeloid cells. | Foundational marker/anatomy evidence. Use to support PVM identification and context-dependent phenotype, not to infer a fixed regulatory/tolerance state. Avoid wording such as 'all' or 'exclusively' unless quoting the original methods precisely. |
| Human, monkey, and mouse CNS; HIV/SIV encephalitis contexts; non-human primate tracer validation (Kim et al., 2006) | CD163 immunostaining; Fluoro-Emerald CSF uptake in monkey; CD14/CD16/HLA-DR blood monocyte markers | Brain perivascular spaces; also meningeal and choroid plexus macrophages; blood monocytes as candidate precursors | CD163 was presented as a PVM marker in normal and viral encephalitic brains and as a marker of potential blood precursors to PVMs; tracer uptake supported perivascular localization in non-human primates. | Useful for PVM marker and precursor context. Do not overstate as 'exclusive' in all disease states; viral encephalitis is a comparator, not evidence for HSV-1 in schizophrenia. |
| Long-term repopulated rhesus macaques after autologous CD34+ hematopoietic stem-cell transplantation (Soulas et al., 2009) | EGFP lineage tracing from CD34+ HSCs; CD163 and CD68 immunostaining | Brain perivascular compartment; blood and bone marrow compartments in the model | Gene-modified CD34+ HSC progeny contributed to turnover of brain PVMs, including CD163+/CD68+ cells. | Supports possible hematopoietic contribution to PVM turnover in primates. CD34 is the progenitor source, not a PVM co-expression marker. Not schizophrenia-specific. |
| Human control and Alzheimer's disease brain (Sasaki et al., 1996) | CD11c; CD68; HLA-DR/MHC-II | Brain perivascular cells | CD11c+ perivascular cells with macrophage/antigen-presentation markers were described in human brain and AD contexts. | Comparator only. CD163 was not assessed. Retain only if labelled as historical PVM/myeloid immunophenotyping; otherwise it may confuse a CD163-focused table. |
| Human multiple sclerosis lesions and control tissue (Zhang et al., 2011) | CD163; HLA-DR/MHC-II; MBP in phagocytic cells | MS lesion rims and active lesions; perivascular and parenchymal myeloid compartments | CD163+ macrophages/microglia accumulated in active MS lesions and showed HLA-DR and myelin-phagocytosis-related features. | Demonstrates context-dependent CD163 expression in CNS inflammation. Useful comparator showing that CD163 is not specific to schizophrenia or a single functional state. |
| Human and experimental TB contexts; peripheral monocyte-to-macrophage differentiation (Lastrucci et al., 2015) | CD16, CD163, MerTK, pSTAT3; IL-10/STAT3 axis; soluble CD163 | Peripheral blood / granuloma-related monocyte-macrophage program | TB was associated with a CD16+CD163+MerTK+pSTAT3+ monocyte-to-macrophage differentiation program linked to disease progression. | Comparator for chronic intracellular inflammatory contexts only. Not CNS or schizophrenia evidence. Use cautiously to justify testable macrophage-state hypotheses, not causality. |

**Note:** This table is a selective contextual summary rather than a systematic review. It does not imply schizophrenia specificity, pathogen carriage, BCG responsiveness, or a fixed macrophage activation state. Direct schizophrenia/post-mortem evidence is separated from foundational PVM marker studies and comparator disease models. Functional interpretation of CD163 requires spatial localization, co-marker panels, molecular validation, disease-specific validation, and appropriate comparison groups. Rows labelled as comparator evidence should not be interpreted as direct evidence for CD163+ PVM involvement in schizophrenia.

**Abbreviations:** AD, Alzheimer disease; BBB, blood-brain barrier; CNS, central nervous system; DLPFC, dorsolateral prefrontal cortex; EGFP, enhanced green fluorescent protein; HLA-DR, human leukocyte antigen-DR; HIV, human immunodeficiency virus; HSC, hematopoietic stem cell; IBA1, ionized calcium-binding adaptor molecule 1; ICAM1, intercellular adhesion molecule 1; MBP, myelin basic protein; MHC-II, major histocompatibility complex class II; MS, multiple sclerosis; PVM, perivascular macrophage; SEZ, subependymal zone; SIV, simian immunodeficiency virus; TB, tuberculosis; TH, tyrosine hydroxylase.
